# Evolutionarily conserved mechanisms for the selection and maintenance of behavioural activity

**DOI:** 10.1098/rstb.2015.0053

**Published:** 2015-12-19

**Authors:** Vincenzo G. Fiore, Raymond J. Dolan, Nicholas J. Strausfeld, Frank Hirth

**Affiliations:** 1 Wellcome Trust Centre for Neuroimaging, Institute of Neurology, University College London, London, UK; 2 Max Planck UCL Centre for Computational Psychiatry and Ageing Research, London, UK; 3 Department of Neuroscience, University of Arizona, Tucson, AZ 85721, USA; 4 Institute of Psychiatry, Psychology & Neuroscience, Department of Basic & Clinical Neuroscience, King's College London, London, UK

**Keywords:** brain evolution, central complex, basal ganglia, sensorimotor representation, attractor state, action selection

## Abstract

Survival and reproduction entail the selection of adaptive behavioural repertoires. This selection manifests as phylogenetically acquired activities that depend on evolved nervous system circuitries. Lorenz and Tinbergen already postulated that heritable behaviours and their reliable performance are specified by genetically determined programs. Here we compare the functional anatomy of the insect central complex and vertebrate basal ganglia to illustrate their role in mediating selection and maintenance of adaptive behaviours. Comparative analyses reveal that central complex and basal ganglia circuitries share comparable lineage relationships within clusters of functionally integrated neurons. These clusters are specified by genetic mechanisms that link birth time and order to their neuronal identities and functions. Their subsequent connections and associated functions are characterized by similar mechanisms that implement dimensionality reduction and transition through attractor states, whereby spatially organized parallel-projecting loops integrate and convey sensorimotor representations that select and maintain behavioural activity. In both taxa, these neural systems are modulated by dopamine signalling that also mediates memory-like processes. The multiplicity of similarities between central complex and basal ganglia suggests evolutionarily conserved computational mechanisms for action selection. We speculate that these may have originated from ancestral ground pattern circuitries present in the brain of the last common ancestor of insects and vertebrates.

## 1. Introduction

Brains of roving animals have evolved to make decisions in response to change in their internal environment based on cues indicative of, for example, nutritional status, and to change in their external environment. Decisions of the first kind involve relatively simple and ancient autonomic circuits that sense and regulate expected variations. Decisions of the second kind involve more complex circuits that serve to detect external events, weigh their saliency and relevance, and decide when and how to act on them [1]. Although different species have evolved elaborate and specific circuits that match their specific ecologies, with respect to the selection of appropriate behaviours brains nevertheless have deep commonalities. A honeybee and a nectar eating bat undertake comparable foraging tasks with central nervous systems of vastly different size and complexity. However, their brains share two fundamental properties. Both can recollect prior actions and select appropriate actions on the basis of present stimuli and recalled associations.

Here we consider evidence that in arthropods and vertebrates, parts of the brain mediating these properties derive from genealogically corresponding circuits, and that without those parts, behavioural activity would be restricted to reflex-like actions. In a cockroach, for example, forward walking can be triggered by touching its abdomen, even after its brain has been disconnected from its ventral nerve cord. But the animal is unable to respond to novel external cues and change its direction [2–5]. A decerebrate cat will walk with a normal gait on a treadmill, but it is unable to respond to novel stimuli that would elicit a change in its gait [6]. In arthropods and vertebrates, selection and maintenance of adaptive motor actions involve more than reflexes. Cerebral ganglia are required for adaptive motor actions, and in both taxa specific regions of the forebrain are required for the selection of such actions. In insects, these regions comprise interconnected centres collectively known as the central complex (CX), which when injected with venoms containing inhibitory transmitter substances cause ataxia and an inability to initiate and mediate voluntary movement [7]. In vertebrates, interconnected centres in the forebrain, known as the basal ganglia (BG), are required for the selection of voluntary behaviours, while their pathological disruption results in ataxia, paralysis and other behavioural deficits as seen in Parkinson's and Huntington's disease [8].

**Figure 1.**
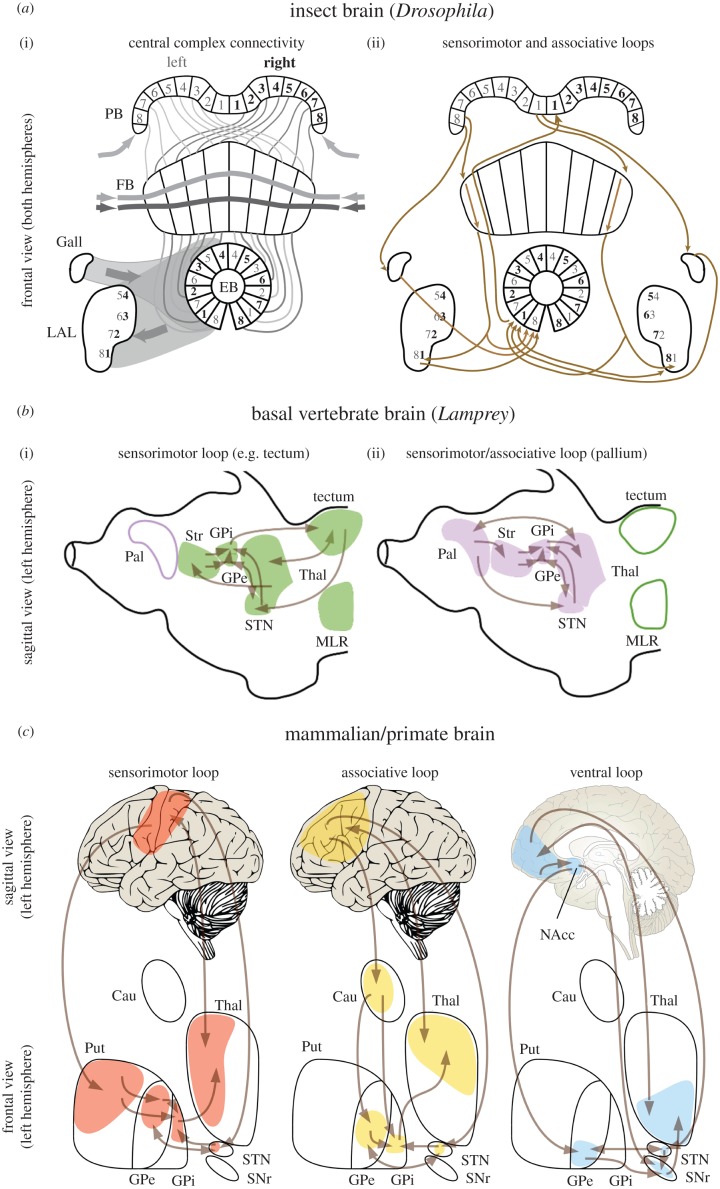
Principal arrangements of the insect central complex, the lamprey and primate basal ganglia and their associated loops. (*a*) (i) Simplified schematic of the central complex (CX) showing connections between the protocerebral bridge (PB), fan-shaped body (FB) and ellipsoid body (EB), along with two satellite neuropils, the Gall and lateral accessory lobe (LAL). The noduli have been omitted. The PB is divided into synaptic modules which, depending on the species, vary between symmetrical arrangements of 9 + 9 (in *Drosophila*, *Mantis religiosa*) and as few as 5 + 5 (*Notonecta*) units. PB modules encode sensory representations (visual/tactile) from eight sectors on each side of the animal's long axis. Left and right representations of this ‘where’ code are relayed across eight modules of the FB such that left and right maps from the PB are compared. Outputs from the FB project into 16 modules of the EB such that modules representing opposite sectors are adjacent. The PB receives numerous inputs, including neurons entering laterally (arrows) carrying high-level information about visual motion direction. Columnar module in the FB are intersect by many dendritic trees and terminals (two shown) that likewise carry synthesized sensory information about complex parameters (‘what’ inputs) as well as a broad palette of modulatory peptides. The EB also receives a variety of inputs (also ‘what’ afferents), such as from the Gall and other satellite neuropils. Different combinations of where and what inputs result in different levels of activity in EB modules. Competing strengths of activity among EB modules result in a few achieving a stable output to the LAL. Further connections link the LAL system to pre-motor descending neurons (not shown). (ii) Comparison between sensorimotor and associative loops in insects (using the anatomy of the *Drosophila* as a general model) and sensorimotor, associative and ventral loops in mammals (using primates and humans in particular as a general model). Spatial organization is highlighted by the presence of numerically ordered modules in PB, FB, EB and LAL. The grey and bold black fonts indicate modules on the left and right side, respectively. (*b*) Anatomical representation of the re-entrant neural circuits characterizing sensorimotor and associative selections in the lamprey, which diverged already 560 Ma from the vertebrate lineages. (i) The first re-entrant neural circuit involves BG, thalamus and areas such as the optic tectum or the MLR, which provide sensorimotor inputs to the striatum via thalamus and receive direct inhibitory output from the BG. (ii) The circuit involves BG, thalamus and pallium, which projects directly towards the striatum and in turn receives mediated (via thalamus) inhibitory output from the BG. (*c*) Sensorimotor, associative and ventral (limbic) loops in mammals, here shown for primates. In the left hemisphere in humans, the different colours highlight the connectivity between separate areas in the cortex and their specific targets in the striatum and thalamus. This parallel partial segregation is maintained throughout the basal ganglia in the GP (globus pallidus), STN and SNr. *Abbreviations*: PB, protocerebral bridge; FB, fan shape body; EB, ellipsoid body; LAL, lateral accessory lobes; Pal, pallium; Str, striatum; MLR, mesencephalic locomotor region; Thal, thalamus; GPe, globus pallidus external segment, GPi, globus pallidus internal segment; STN, subthalamic nucleus, SNr, substantia nigra pars reticulata; Cau, caudate; NAcc, nucleus accumbens; Put, putamen.

The insect CX is composed of five discrete interconnected midline neuropils in the anterior-most segment of the brain (protocerebrum). These are the protocerebral bridge (PB), fan-shaped body (FB), ellipsoid body (EB), the paired noduli (NO) and the paired lateral accessory lobes (LAL) [9,10] (figure 1*a*). The vertebrate BG consists of an arrangement of basal forebrain nuclei that includes the striatum (which in primates consists of caudate, putamen and ventral striatum, including nucleus accumbens), the internal and external domains of the globus pallidus (GPi and GPe, respectively), the subthalamic nucleus (STN), and the substantia nigra pars reticulata (SNr) (figure 1*b,c*) [11].

Both the CX and the BG share extensive similarities in their heritable ontogeny and behavioural performance, and previous analyses have identified multiple correspondences between them. These include their embryological derivation and orthologous genetic specification, neural architectures, neurochemical attributes and physiological properties as well as behavioural outcomes of neuronal activity, including pathologies [12,13]. Together these correspondences imply a common ancestral origin of circuits that have diverged over a timespan of more than 540 Myr to provide the insect CX and the vertebrate BG. Here we extend this comparative analysis and identify common principles underlying the functional anatomy of the CX, the BG and their associated circuits. Our analysis suggests that evolutionarily corresponding computational mechanisms underlie the selection and maintenance of adaptive behaviour in insects and vertebrates.

## 2. Clonal unit architecture and functional compartmentalization

Both the CX and BG substructures derive from neural stem cells of the basal forebrain [12] that generate lineage-related sister cells. Here we first consider the BG. Earlier studies in rats showed that injection of [^3^H] thymidine, as well as retroviral-mediated gene transfer, marked the progeny of individual progenitors at different times of embryonic development that in turn identified the lineage relationship of striatal neurons [14,15]. These studies also revealed that neuronal birth dates define the segregation of striatal neurons, with clonal units either populating striosomes or matrix [14], thus contributing to the formation of functionally distinct compartments in the striatum [16,17]. More recent studies using lineage analyses of genetically modified mice expressing a traceable marker, identified that Nkx2.1-expressing progenitor cells of the embryonic subpallium generate distinct subpopulations of interneurons of the striatum as well as projection neurons of the globus pallidus [18], including cholinergic and GABAergic interneurons [19]. Moreover, analysis of mouse mutants revealed a Dlx1&2-dependent sequence of transcription factor activity required for the specification of striatal neurons [20], suggesting that neuronal cell fate within BG substructures is determined by lineage relationship and their birth time/order, which are mediated by combinatorial codes of selector gene functions.

Comparable regulatory programs for the spatio-temporal specification of neuronal fates have been identified for the insect CX. Using genetically marked neural stem cells, called neuroblasts (NBs), studies of *Drosophila* reveal that the CX derives from a limited number of NBs whose lineage-specific progeny constitute specific columns, layers and modules of CX substructures [21–25], thus mediating the formation of functionally distinct compartments comparable to the neural organization in the vertebrate BG. Such studies, which use mosaic analysis with a repressible cell marker (MARCM), also showed that lineage-specific progeny acquire a neuronal identity owing to their birth time and order directed by the spatio-temporal activity of selector genes. For example, a detailed analysis of a group of six neurons innervating layers of the EB and LAL in a cell type-specific manner demonstrated that they are generated in an invariant contiguous order from one single progenitor cell [26]. This process requires Chinmo, a *broad complex*, *tramtrack*, *bric-a-brac* (BTB)-zinc finger nuclear protein, which selectively regulates the third temporal identity among the six neurons [26]. These data provide evidence that CX substructures share common lineages in functionally organized groups, mediated by genetic mechanisms that link birth time and order to neuronal identity and function. Given that comparable mechanisms underlie the formation of BG nuclei, together these findings suggest that genetically encoded clonal units, also referred to as ontogenetic clones [27], are evolutionarily conserved cytoarchitectonic modules underlying the heritable ontogeny and reliable performance of BG and CX circuits.

## 3. Parallel-projecting, partially segregated circuits for sensorimotor and associative representations

Previous comparative analyses revealed that the CX and BG have executive control over comparable behaviours, and that their development or disease-related dysfunction can lead to homologous pathologies, including movement disorders, such as Parkinsonism, as well as neuropsychiatric disorders like schizophrenia, essentially affecting goal-directed behaviour and habitual control [12]. To identify the underlying computational mechanisms, we further examined the functional anatomy of CX and BG circuitry.

### (a) Central complex circuitry

Histological, immunocytochemical and clonal analyses reveal that the functional anatomy of the CX is built on three structural principles: columns, modules and layers [21,23–25,28,29]. Columnar projection neurons are those that extend through the depths of three successive centres (neuropils): the PB, FB and EB, which are themselves further divided into discrete domains (figure 1*a*(i)). Columnar neurons are reiterated across the lateral extent of these neuropils, which are anatomically subdivided into modules. Each module usually contains the same set of columnar neurons, certain of which encode spatial information about sensory events surrounding the organism. Such events include tactile [30] and visual cues [5,31–34]. Modules are intersected by layered arrangements of wide-field dendrites and terminals, in particular, neurons carrying neuroactive peptides or that relay information indirectly from learning and memory processing regions, such as the mushroom bodies [34]. Their terminals contribute to the stratification of the FB and EB where they intersect columnar neurons disposed in the repeated arrangement of modules across the CX [21–25,28,29]. In general, this arrangement provides a substrate for multisensory space to be mapped across the CX, whereby a representation of a defined segment of sensory space is functionally represented in each module.

In the *Drosophila* PB, there are 18 such modules. Nine each side of the midline, represent one-half of the sensory hemisphere [21–25]. Axons from each half of the PB distribute across the entire width of the FB, which is the next level of the CX. This arrangement ensures that, within the FB, corresponding loci from the two halves of the sensory hemisphere interact [28,29]. The layered organization of efferents to the FB provide high-level information about sensory events irrespective of location [35]. CX modules are thus disposed to compare encoded events across the entire representation of sensory space thereby permitting, in principle, global assessment of stimuli. Interactions between modules and strata, together with modulatory aminergic inputs extending across modules (mainly dopamine, and also serotonin (5-HT) and octopamine), ultimately shape the output from the CX to neuropils that interact with descending channels carrying information from the brain to sensorimotor circuits in thoracic and abdominal ganglia [5]. Recordings and experiments, in which lesions or local stimulation result in specific behavioural defects, suggest that it is the interactions among modules that determine the nature of the expression of motor actions [36]. However, for this to happen they must provide outputs to at least one computational layer that determines which of many concomitant sensory signals are the most relevant in terms of whatever behaviour in which the insect is engaged. Such outputs from the FB supply the EB, the deepest level of the CX, and it is at this level that arrays of tangential neurons provide reciprocal connexions among modules allowing such decisions to be made [37] and relayed to the bilateral arranged LALs and their connections to descending channels (figure 1*a*).

### (b) Central complex circuitry in sensorimotor transformation

At least three sensory modalities (visual, mechanosensory, temperature) are relayed to the PB and FB in the form of highly structured codes [10]. Each represents one of many presumably simultaneous sensory events as well as recollections that must be assessed for their most probable adaptive value. The computational role of the EB is to determine what within this incoming stream of data is best translated as possible motor actions. It is proposed that outputs from the EB serve to gate those parts of the LAL that have executive control of the activity of descending channels, the role of which is to appropriately modify local sensory-motor circuits to accomplish complex motor actions. It is assumed that reafferent copy from the motor output, and the LAL to the CX, closes the feedback loop (see below).

This conceptual framework [12,38] is supported by a number of studies using targeted inactivation of the CX that identify specific regions and neuronal subtypes as essential anatomical substrates for the selection and maintenance of behavioural activity, ranging from courtship and orientation behaviours, to visual memory and place learning, as well as attention, arousal and decision-making [39–51]. These data suggest that specific sensorimotor and associative representations are integrated and processed in partially segregated neural loops of the CX, which is best illustrated by the functional properties of the EB.

The EB is composed of populations of nerve cells that have been described in several insect species [52]. In *Drosophila*, these so-called ring neurons (R-neurons) are classified into at least four subtypes (R1–R4) based on their morphology, synaptic organization and terminal arborizations that define concentric layers of the EB neuropil [24,25,29]. Previous studies showed that EB ring neurons of the R3 subtype mediate selection between opposing visual cues for orientation [53] and spatial memory formation [42]; whereas EB R1 neurons were shown to process place learning in a heat maze where flies had to find a hidden cool place in an otherwise noxious 36°C environment [48]. EB R2/R4 neurons were found to mediate visual pattern memory in a flight simulator where a fly could choose its flight direction relative to visual patterns [43]. The same EB R-neuron subtype proved to be involved in ethanol sensitivity and tolerance [54], and ethanol-induced locomotion [46], where flies were given the choice for ethanol intake. The same R2/R4 neurons have been found to regulate repetitive startle-induced arousal when flies were exposed to mechanical stimulation by repeated air puffs [44]. A comparable organization into partially segregated functional units has been observed for different layers of the FB involved in locomotion, visual orientation and memory (e.g. [35,39,43,51]). Moreover, recent connectomics-based information flow analysis suggests that this partially segregated organization not only applies within each CX substructure but also to columnar projections across them [55]. Given its structural organization into columns, layers and modules, these findings suggest a functional anatomy of the CX whereby parallel-projecting, partially segregated loops integrate and convey sensorimotor and associative representations for the coordination and control of adaptive behaviour (figure 1*a*).

### (c) Basal ganglia circuitry

A comparable structural and functional organization in the basal ganglia also characterizes two overlapping classes of recurrent loops, so-called re-entrant circuits, that have been identified across vertebrate species [11,56,57]. Studies on lamprey (figure 1*b*), a jawless fish closer to the base of the vertebrate lineage than any other extant vertebrate species, highlight the presence of both classes of circuits and their extensive similarities with the equivalent neural structures in mammals (cf. figure 1*c*).

In the first class of these circuits, the BG establish a series of partially segregated re-entrant circuitries involving distinct parts of the thalamus and brainstem motor centres such as the optic tectum (superior colliculus in mammals), or the mesencephalic locomotor region (MLR) [57–62]. Each sensorimotor input source projects, via the thalamus, towards separate parts of the input nuclei of the BG (striatum and STN) and each in turn is directly inhibited by specific sub-domains of the BG output nuclei (GPi/SNr). This partial segregation enables the re-entrant circuits to process information separately, displaying high functional specialization in controlling motor programs associated with specific sensory stimuli (e.g. the optic tectum conjunctly controls visual stimuli and oculomotor activity) [60].

For the second class of circuits, a similar re-entrant loop involves BG, thalamus and pallium (cortex in mammals) [63]. Compared with the first class of re-entrant circuits, the pallium directly projects towards the striatum and the STN, and is indirectly inhibited by the output of the BG via the thalamus [64]. The partial overlap characterizing these two classes of loops, jointly with dopaminergic innervation [65], is thought to allow the pallium to function as both an associative system and a bridge for the exchange of sensorimotor information among parallel loops. In mammals, this function is exemplified by the extensive development of the cortex, which takes the place of the pallium in the re-entrant circuit formed with the BG and thalamus, enabling more elaborated motor control for voluntary movements. Studies in primates and rodents have identified the presence, alongside the subcortical re-entrant circuits [60], of three major re-entrant striato-thalamo-cortical circuits to control and select sensorimotor, associative and limbic information (figure 1*c*) [66,67].

### (d) Basal ganglia circuitry in sensorimotor transformation

Both classes of thalamo-striatal loops exploit the structure and information processing of the BG to realize the same function of selection via gating [60,68–71], adjusting for different input and output nuclei. The input reaching the BG is spatially and somatotopically organized to preserve information about the input stimuli [67]. The presence of channels within the BG allows these nuclei to process separately each element in the input, maintaining, amplifying or suppressing them in their path towards the output nuclei of the BG [69]. Selection and maintenance of behaviour in the BG relies on the converging signal conveyed by three pathways into the gating systems of the GPi and SNr (figure 1*b,c*). These are called the *direct*, *indirect* and *hyperdirect* pathways, the first two of which originate in the striatum and the third originating in the cortex [66]. Direct and indirect pathways are characterized by two distinct populations of GABAergic ‘medium spiny neurons (MSN)’ that are distinguished by their morphologies and the expression of distinct dopamine receptor subtypes [8,72].

The *direct pathway* consists of direct parallel inhibitory circuits originating from striatal MSNs characterized by dopamine D1 (excitatory) receptors and projecting towards the GABAergic output nuclei of the BG (either GPi or SNr). The *indirect pathway* originates from striatal MSNs characterized by dopamine D2 (inhibitory) receptors and reaches the BG output nuclei via GPe. The GPe itself projects inhibitory connections to both GPi and SNr. Finally, the *hyperdirect pathway* bypasses the striatum and connects the cortex directly to the STN, which then sends glutamatergic projections to the GPi (figure 1*c*) [73].

## 4. Dimensionality reduction via sensory integration

The connectivity within PB–FB–EB–LAL and thalamo-striatal loops realizes another shared and essential feature of the CX and BG, termed dimensionality reduction. Dimensionality reduction describes the processing of inputs from a high-dimensional data space to a lower dimensional space, which in the brain entails the compression of information encoded by a large neuronal population to a smaller one (as is the case, for instance, for visual and olfactory inputs). Its efficiency is measured by the ability to preserve information from the original data space, which can be the combined activity of a population of neurons that encode specific variables such as the angle of a looming object, or the angle of limb movement [74].

In both CX and BG, dimensionality reduction is achieved by sensorimotor and associative loops, which receive information from basically all regions of the brain. None of the CX and BG neuropil/input nuclei are directly connected to brain regions that encode sensory data. Thus, processed input is integrated and conveyed within loops whereby dimensional space is significantly reduced. In the case of the rat BG, the cortico-striatal reduction is 10 : 1, followed by a 1000 : 1 reduction between striatum and the GPi and SNr output nuclei [74].

### (a) Dimensionality reduction in the basal ganglia

In mammals, the ventral part of the striatum acts as a nexus for the integration of information received from the amygdala (object-related values), hippocampus (spatial-related value and novelty) and prefrontal and orbitrofrontal cortices (future outcomes) [75,76]. Processed information in the ventral loop converges also on the sensorimotor loop such that this information flow is spatially organized and is added to information conveyed from sensory areas of the motor cortex (feed-forward control). Here the process of selection is repeated, integrating sensory signals from different modalities [77], thereby triggering an appropriate motor response that is once again provided to the ventral loop as a reafferent (feedback) signal. Comparable feed-forward and feedback systems involved in motor control occur among the subpallial thalamo-striatal loops where this bidirectional information flow enables, for instance, coordination of visuo-motor information of head orientation and body postures [78]. The significance of dimensionality reduction [74] is nicely illustrated in freely moving rats. While exploring a cage, their striatal neurons’ firing rates encode both spatial and behavioural features at the same time [79], including head movement velocity [80], whereas the firing rates of specific classes of SNr neurons encode a cartesian *x* or *y* coordinate of the position vector [81].

### (b) Dimensionality reduction in the central complex

Similar to its vertebrate counterpart, the input layers of the EB function as a nexus for heterogeneous sensory information (visual, tactile, haptic, gravitational). In *Drosophila,* incoming processed sensory inputs covering large areas of sensory space are integrated and conveyed by a population of columnar neurons and EB neurons, as has been shown for polarized light information that is assumed for navigation [32,41,45], for features and orientations of moving objects [34,51] and for the position of an object within the visual field in relation to the animal's own body position [34]. Thus, high-dimensional data space (e.g. a moving object across the visual field) is reduced to a lower dimensional space (e.g. the activity of columnar neurons), which is the case for both BG and CX. Accordingly, the stimulus-related neural activity of channels (for the BG) or columns (for the CX) has been shown to encode, for instance, a specific action or goal [82], movement velocity [80], space coordinates or body orientation [34,81]. Dimensionality reduction thus integrates sensory stimuli and behavioural repertoires, and therefore aids in the selection of motor actions, and the coordination and control of behavioural activity.

In addition to dimensionality reduction and sensorimotor integration, stimulus-related neural activities of channels (for the BG) or columns (for the CX) are thought to become reference signals that are coupled with the transformation of sensory input. Coupling enables the association of a behavioural selection with its immediate perceivable consequences in the environment [83]. Such feedback loops establish a neural gain [84] that can either amplify or suppress the signals represented in the channels or columns and thus corroborate or diminish a behavioural selection.

### (c) Nonlinear dynamics and attractor states

**Figure 2.**
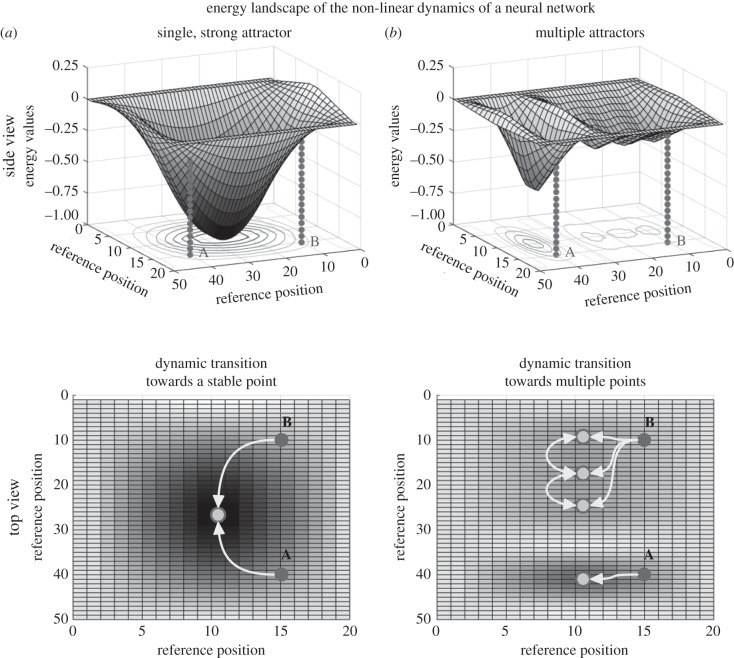
Energy landscape illustrating nonlinear dynamics of a hypothetical neural network. The nonlinear dynamics of neural networks can be described in energy landscapes, where any starting condition of the system will cause the network to evolve and change its activity towards the closest low energy state, the ‘attractor’. (*a*) In this first example, two different starting conditions (A and B), initially located in a high energy position, reach the bottom of the basin offered by a single vast attractor state. The initial considerable differences in the input are ignored by the system which, via state transitions, reach the same stable point. (*b*) In this second example, the same starting conditions (A and B) in a similar high energy starting position are characterized by very different state transitions. State transition for A results in reaching the closest low energy state of a small attractor state, if compared with the attractor represented in (*a*). Conversely, state transition for B causes movement towards a very shallow area, where minor perturbation (e.g. by noise) can result in reaching any of three different unstable points and further perturbation can trigger frequent switch among these weak attractors. The same illustrative heat maps of the energy landscapes of an arbitrary network are depicted twice using different perspectives to highlight its three-dimensional features.

In computational terms, re-entrant neural networks exhibit nonlinear input–output transformations. These dynamics can be described as complex energy landscapes, characterized by the presence of attractor states (figure 2) [85,86]. The spatially organized loops generate a series of parallel feedback microcircuits that compete against one another for the control of the activation pattern of the system [37,87–89]. Each channel is characterized by a specific neural gain, which generates an attractor in the energy landscape. Here, the stronger the gain characterizing the self-sustaining channel, the steeper and larger the attractor. When a sensory input perturbs the system, thereby activating a pattern of activity, it triggers a transition towards the closest stable point (figure 2). Once stable, the system maintains its activity until further perturbed.

In general, depending on the steepness of an attractor, a transition (and hence the appraisal of the sensory input) may take a very short or long time. At the bottom of the attractor basin (low energy state), the transition phase ends, competition with other potential attractors terminates and a behavioural selection is triggered so that each attractor state in the sensorimotor loop is associated with a unique motor response [34,81,90,91].

## 5. Neural mechanisms and computations for action selection

Adaptive behaviour can be parsed into a number of sub-functions, including the integration of multiple sensory stimuli (dimensionality reduction), the suppression of noise and irrelevant competing signals, and the detection and selection of the most salient stimulus in reference to the internal state of the agent. These operations must show a certain degree of context specificity. First, they enable switching between a configuration that needs quick adaptation to changes in the sensory input and another configuration that affords maintenance of a selection (persistent behaviour) [88,92]. Second, the motor selections that are eventually triggered as a response to the combined effect of the internal state condition and perturbation by sensory stimuli have to be updated on the basis of previous experience that exploits knowledge about the environment and predictive cues.

### (a) Basal ganglia direct and indirect pathway activity in action selection and maintenance

Within the respective loops, EB and BG are ideally positioned for such selections, with their location being downstream of sensory inputs and upstream of motor outputs. For the vertebrate BG, a wealth of studies suggest that an interaction among the three putative pathways (direct, indirect and hyperdirect) determines the selection process among the different channels and attractor states [8,93]. For instance, optogenetic manipulations show that direct pathway stimulation in the sensorimotor loop facilitates behavioural activity, whereas indirect pathway stimulation decreases motor action [94], both of which are cooperatively active during voluntary movement [95,96]. These data support computational hypotheses suggesting that direct pathway activity is coherent with the gain of the loops, strengthening attractor states and facilitating the attainment of a stable point, when carrying out a selection. By contrast, indirect and hyperdirect pathway activities interfere with gain, resulting either in increased instability and longer transition phases, owing to shallow attractors, thereby diminishing the ability to carry out a selection [68–70,97] or in the generation of cyclic attractors [90]. Indeed, BG oscillations have been considered to emerge from pathological dysfunctions under low dopaminergic conditions, causing motor disabilities such as tremor (e.g. in Parkinson's disease) [97]. More recently, optogenetic manipulation and recordings in mice show that the indirect pathway actively contributes to action initiation and the control of contraversive movement, [95,98], a type of movement involving turns to the left or to the right that are controlled by the opposite hemisphere of the brain. This new understanding calls for further amendments to the standard computational model of BG motor control [8], suggesting that the indirect pathway may also play a fundamental role in controlling the frequency of contraversive oscillatory motor selections [90].

### (b) The central complex ellipsoid body circuitry in action selection and maintenance

**Figure 3.**
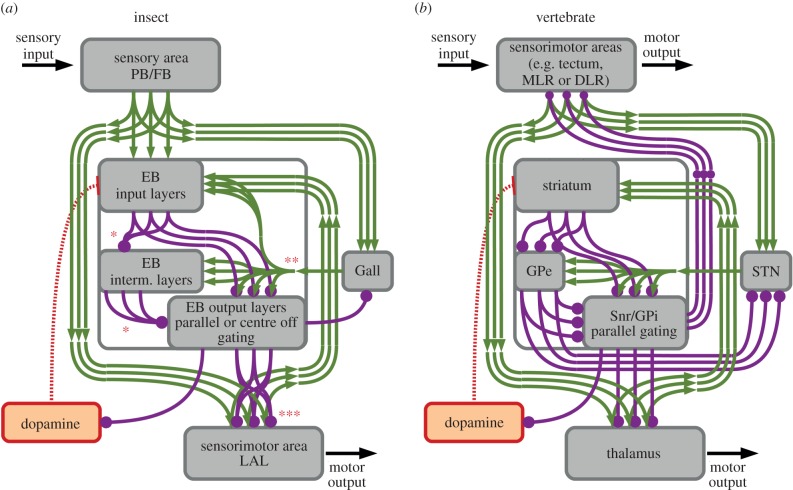
Comparison of action selection circuitries in the insect central complex and vertebrate basal ganglia. Schematic representation of sensorimotor loop, underlying action selection in the (*a*) insect central complex and (*b*) vertebrate basal ganglia, together with modulatory dopaminergic loop. In both insects and vertebrates, the sensorimotor loop is responsible for processing multiple sensory stimuli that are somatotopically organized. Information about the selections performed is projected backwards via feedback excitatory parallel connections. The loop enables the creation of attractor states, which in turn result in noise cancellation, the detection and selection of the most salient stimulus (winner-take-all functionality) and rapid switching among attractor states thereby adapting to changes in the environment. The dopamine loop, via differential dopamine release, modulates gain of the sensorimotor loop, which in turn amplifies or suppresses the information entering the EB in insects and the BG in vertebrates. The dopaminergic loop is responsible for long-term memory formation (via reinforcement learning) and dynamic alteration of attractor states resulting via maintenance of sensory-driven flexibility (short-term memory). The internal wiring of CX and BG only allows a partial comparison as the information in insects is as yet incomplete. In particular, (*) directionality and the presence of parallel connectivity within the EB is a likely explanation of recent behavioural data, but the exact composition of these plausible internal pathways is not yet known. (**) The Gall, connected between PB, EB and LAL, projects towards the EB, but the specific targets in terms of EB layers have to be defined (e.g. the Gall might project towards the output layers only, as for STN in vertebrates). Finally, (***) the gating function performed by the EB may be realized via either parallel or centre-off inhibitory connections: the presence of a directionality in the EB strongly suggests the presence of parallel gating as it would match the computational requirements for the system. *Abbreviations*: PB, protocerebral bridge; FB, fan shape body; EB, ellipsoid body; interm. layers, intermediate layers; LAL, lateral accessory lobes; MLR, mesencephalic locomotor region; DLR, diencephalic locomotor region; GPe, globus pallidus external segment, GPi, globus pallidus internal segment; STN, subthalamic nucleus, SNr, substantia nigra pars reticulata.

In the insect CX, the selection of behavioural activity is mediated by the EB, which largely consists of a GABAergic structure receiving spatially organized sensory information from the PB and FB conveyed towards the EB via columnar projections [21–25,29]. The LAL also receives parallel and spatially organized sensory information from both the PB and FB and is directly connected to motor areas, therefore concatenating functional elements that in vertebrates are represented by the thalamus and the motor area subjected to the gating process (figure 3*a*). As for the subcortical thalamo-striatal loops, whereby the BG directly gates several motor command regions [57], the neural architecture of the columnar organization involving EB and LAL points to the existence of two sensorimotor circuits replicating the same connectome [25] that putatively subserves the selection of different types of motor responses and sensorimotor associations. Finally, the LAL also projects segregated information towards the EB, realizing the feedback required for the reference signal and the input–output coupling (figure 3*a*).

Consistent with this interpretation, we identified lateral inhibition within EB layers and observed that over-activation or inhibition of specific EB layers results in significantly different effects on motor behaviour [37]. Based on these observations, we hypothesize that the EB expresses a certain degree of directionality, which in turn points to the presence of specialized input and output layers likely to be found among EB R1–R4 and the recently identified posterior (P) layer [24]. This hypothesis is supported by the presence of a small accessory neuropil to the CX-LAL called the ‘Gall’ [22,25]. Based on its connectivity between PB, EB and projections towards the EB (figures 1*a* and 3*a*), the Gall might computationally play a role comparable to that of the STN, which exerts regulating tonic activity on the inner and output nuclei of the GP and SNr. These internal nuclei require a strong, tonic excitatory input to balance the lateral and afferent inhibitions derived from the striatum (for the BG) and from the other layers of the EB (in the CX).

### (c) Action selections as transitions through attractor states

Computationally, selections are achieved by means of state transitions among attractors, whose shapes (e.g. steepness and surface dimension) are directly determined by the gain of each channel or column in sensorimotor and associative loops. The presence of multiple attractors in both insects and vertebrates is the key requirement for a winner-take-all mechanism that suppresses competing behavioural responses and avoids the risk of triggering multiple incompatible action selections. Multiple attractors guarantee the selection of the most appropriate response to a given salience. Conversely, oscillatory selections require the presence of cyclic attractors, which allow a single sensory input to be processed in a periodic transition between states, in a pendulum-like activity.

The BG neural architecture is compatible with both types of attractors, with a key role of the direct pathway for stable attractors and their maintenance, and a key role for the indirect pathway (involving the direct connection between the GPe and either the GPi or the SNr) for oscillatory attractors [90]. It is reasonable to assume that the EB may play a similar role, sharing with the BG the ability to control both tonic steady action selections and patterns of alternating (slow oscillatory) motor selections to regulate rhythmic movements. In support of this notion, *Drosophila* mutants with structural defects of the FB and EB are unable to walk straight, with their motor behaviour characterized by circling and severely reduced speed [39,99], suggesting that alternating motor selections required for straight walking are impaired.

In both vertebrates and insects, the presence of such differential activity requires an internal mediator to modulate the switch from a condition favouring stable selections and another favouring instability or patterns of selections. In vertebrates, this role is played by dopamine through its differential effects on direct and indirect pathway activities in the BG [95,96,100].

## 6. Dopaminergic control: short- and long-term alteration of action selections

The significance of a stimulus is weighed with reference to previous (stored) experience. Dopamine has been identified as the principal modulator for this computational task, which applies to both CX and thalamo-striatal loops [12,65]. Both EB in the CX and GPi/SNr in the BG convey inhibitory signals towards a dopaminergic area. In turn, functioning of the loops is thought to be highly affected by the fast (phasic) and slow (tonic) fluctuations of dopamine release. The presence, causes and dynamics of dopaminergic bursts match those required by prediction error signals, supporting the hypothesis that phasic dopamine controls learning and memory formation in vertebrates [101,102] and insects [103,104]. In terms of neural connectivity, phasic dopamine bursts allow the system to change the strength of the connections conveying sensory information towards the EB or the BG. In turn, this alteration changes the way a sensory stimulus will be weighed in the future [105], affecting the probability that the motor action that resulted in the dopamine bursts will be selected again, thereby realizing reinforcement learning (e.g. [87,89]). By a long-lasting process that strengthens or weakens the gain established in each channel or column of the sensorimotor loops, the energy landscape associated with the neural system is constantly updated. After a few trials causing dopamine bursts, sensory inputs and reference action signals are thought to be coupled with a learning process that results in stronger gain in the respective channels or columns and therefore wider and steeper attractors. Thus, the shape and the steepness of the attractors are ‘storing’ information about the most positive or negative motor responses (i.e. causing phasic dopamine bursts and learning) to a perceived input [106]. For instance, over-trained neural systems exhibiting habits [107] are characterized by vast and steep attractor states in their energy landscapes. In these systems, even a partial identification of the over-trained stimulus makes the system fall into the associated attractor, triggering the learned action selection.

Slow tonic dopamine influences also impact on the attractor state landscape of control loops, though their effect is limited in time and results in phenomena resembling short-term memory [42,108]. Fluctuations in tonic dopamine do not alter the strength of the projections conveying sensory input information towards the EB or BG. On the contrary, tonic dopamine temporarily amplifies (in the case of D1 receptors) or compresses (in case of D2 receptors) the strength of the signal conveyed. In the sensorimotor loop, this modulation alters the gain of all channels affected by such fluctuations in dopamine parameters, contrary to channel-specific alterations characterizing long-term reinforcement learning. As a consequence, the effect is not to favour a single sensorimotor response, but rather to make the whole system generally more stable (in case of amplification), or unstable (in case of compression). The first condition allows for maintenance of a configuration pattern and a selection despite significant alteration of the sensory input, whereas the second condition drives the system to more frequent changes in selections, which can now be caused by even minor changes in the sensory input [88].

## 7. Summary and conclusion

Our comparative analysis identifies extensive correspondences of the functional anatomy of the CX and BG and their associated loop architecture (figure 3). The CX and BG share lineage relationships in functionally organized groups (ontogenetic clones), mediated by genetic mechanisms that link birth time and order to neuronal identities and functions. Similarly, the connectivity of these circuitries and the associated functionality are characterized by dimensionality reduction and attractor states whereby spatially organized parallel-projecting, partially segregated and yet interconnected loops integrate and convey sensorimotor representations for the selection and maintenance of behavioural activity. The underlying action selection mechanisms include integration of multiple sensory stimuli, the suppression of noise and less relevant competing signals, and the detection and selection of the most salient stimulus, while simultaneously suppressing competing behavioural responses in order to prevent multiple, incompatible, selections and motor outputs. The resulting selection of a single sensorimotor response is modulated by differential dopamine signalling that can mediate short- and longer-term maintenance, and thus short and longer-term memory. Given the extensive similarities in their origin, genetic specification, circuit architecture and behavioural output [12], the described sensorimotor circuits epitomize Lorenz & Tinbergen's postulate [109] that phylogenetically acquired behavioural activity relies on the physiological function of CNS substructures, whose heritable ontogeny and reliable performance depend on a genetically determined program. Thus, the multiplicity of similarities described here identifies conserved computational mechanisms underlying action selection, suggesting a shared evolutionary origin of the CX and BG.

## References

[1] Stirling P, Laughlin S 2015 Principles of neural design. London, UK: MIT Press.

[2] Schaefer PL, Ritzmann RE 2001 Descending influences on escape behavior and motor pattern in the cockroach. J. Neurobiol. 49, 9–28. (10.1002/neu.1062) 11536194

[3] Ritzmann RE, Pollack AJ, Archinal J, Ridgel AL, Quinn RD 2005 Descending control of body attitude in the cockroach *Blaberus discoidalis* and its role in incline climbing. J. Comp. Physiol. A 191, 253–264. (10.1007/s00359-004-0537-0) 15309482

[4] Ridgel AL, Ritzmann RE 2005 Effects of neck and circumoesophageal connective lesions on posture and locomotion in the cockroach. J. Comp. Physiol. A 191, 559–573. (10.1007/s00359-005-0621-0) 15864596

[5] Ridgel AL, Alexander BE, Ritzmann RE 2007 Descending control of turning behavior in the cockroach, *Blaberus discoidalis*. J. Comp. Physiol. A 193, 385–402. (10.1007/s00359-006-0193-7) 17123086

[6] Whelan PJ 1996 Control of locomotion in the decerebrate cat. Prog. Neurobiol. 49, 481–515. (10.1016/0301-0082(96)00028-7) 8895997

[7] Kaiser M, Libersat F 2015 The role of the cerebral ganglia in the venom-induced behavioral manipulation of cockroaches stung by the parasitoid jewel wasp. J. Exp. Biol. 218, 1022–1027. (10.1242/jeb.116491) 25687435

[8] Nelson AB, Kreitzer AC 2014 Reassessing models of basal ganglia function and dysfunction. Annu. Rev. Neurosci. 37, 117–135. (10.1146/annurev-neuro-071013-013916) 25032493 PMC4416475

[9] Ito K et al. 2014 A systematic nomenclature for the insect brain. Neuron 81, 755–765. (10.1016/j.neuron.2013.12.017) 24559671

[10] Pfeiffer K, Homberg U 2014 Organization and functional roles of the central complex in the insect brain. Annu. Rev. Entomol. 59, 165–184. (10.1146/annurev-ento-011613-162031) 24160424

[11] Stephenson-Jones M, Samuelsson E, Ericsson J, Robertson B, Grillner S 2011 Evolutionary conservation of the basal ganglia as a common vertebrate mechanism for action selection. Curr. Biol. 21, 1081–1091. (10.1016/j.cub.2011.05.001) 21700460

[12] Strausfeld NJ, Hirth F 2013 Deep homology of arthropod central complex and vertebrate basal ganglia. Science 340, 157–161. (10.1126/science.1231828) 23580521

[13] Strausfeld NJ, Hirth F 2013 Homology versus convergence in resolving transphyletic correspondences of brain organization. Brain. Behav. Evol. 82, 215–219. (10.1159/000356102) 24296550

[14] van der Kooy D, Fishell G 1987 Neuronal birthdate underlies the development of striatal compartments. Brain Res. 401, 155–161. (10.1016/0006-8993(87)91176-0) 3028569

[15] Krushel LA, Johnston JG, Fishell G, Tibshirani R, van der Kooy D 1993 Spatially localized neuronal cell lineages in the developing mammalian forebrain. Neuroscience 53, 1035–1047. (10.1016/0306-4522(93)90487-Z) 7685067

[16] Crittenden JR, Graybiel AM 2011 Basal ganglia disorders associated with imbalances in the striatal striosome and matrix compartments. Front. Neuroanat. 5, 59 (10.3389/fnana.2011.00059) 21941467 PMC3171104

[17] Lanciego JL, Luquin N, Obeso JA 2012 Functional neuroanatomy of the basal ganglia. Cold Spring Harb. Perspect. Med. 2, a009621 (10.1101/cshperspect.a009621) 23071379 PMC3543080

[18] Xu Q, Tam M, Anderson SA 2008 Fate mapping Nkx2.1-lineage cells in the mouse telencephalon. J. Comp. Neurol. 506, 16–29. (10.1002/cne.21529) 17990269

[19] Flandin P, Kimura S, Rubinstein JLR 2010 The progenitor zone of the ventral medial ganglionic eminence requires Nkx2-1 to generate most of the globus pallidus but few neocortical interneurons. J. Neurosci. 30, 2812–2823. (10.1523/JNEUROSCI.4228-09.2010) 20181579 PMC2865856

[20] McKinsey GL, Lindtner S, Trzcinski B, Visel A, Pennacchio LA, Huylebroeck D, Higashi Y, Rubenstein JL 2013 *Dlx1&2*-dependent expression of *Zfhx1b* (*Sip1, Zeb2*) regulates the fate switch between cortical and striatal interneurons. Neuron 77, 83–98. (10.1016/j.neuron.2012.11.035) 23312518 PMC3547499

[21] Ito K, Awasaki T 2008 Clonal unit architecture of the adult fly brain. Adv. Exp. Med. Biol. 628, 137–158. (10.1007/978-0-387-78261-4_9) 18683643

[22] Ito M, Masuda N, Shinomiya K, Endo K, Ito K 2013 Systematic analysis of neural projections reveals clonal composition of the *Drosophila* brain. Curr. Biol. 23, 644–655. (10.1016/j.cub.2013.03.015) 23541729

[23] Yang JS, Awasaki T, Yu HH, He Y, Ding P, Kao JC, Lee T 2013 Diverse neuronal lineages make stereotyped contributions to the *Drosophila* locomotor control center, the central complex. J. Comp. Neurol. 521, 2645–2662. (10.1002/cne.23339) 23696496 PMC3902843

[24] Lin CY, Chuang CC, Hua TE, Chen CC, Dickson BJ, Greenspan RJ, Chiang AS 2013 A comprehensive wiring diagram of the protocerebral bridge for visual information processing in the *Drosophila* brain. Cell Rep. 3, 1739–1753. (10.1016/j.celrep.2013.04.022) 23707064

[25] Wolff T, Iyer NA, Rubin GM 2015 Neuroarchitecture and neuroanatomy of the *Drosophila* central complex: A GAL4-based dissection of protocerebral bridge neurons and circuits. J. Comp. Neurol. 523, 997–1037. (10.1002/cne.23705) 25380328 PMC4407839

[26] Yu H, Chen C, Shi L, Huang Y, Lee T 2009 Twin-spot MARCM to reveal the developmental origin and identity of neurons. Nat. Neurosci. 12, 947–953. (10.1038/nn.2345) 19525942 PMC2701974

[27] Furukuo-Tokunaga K, Ludlow ZN, Hirth F 2012 *Drosophila* memory circuits. In Memory mechanisms in health and disease (ed. Giese KP), pp. 269–306. Singapore: World Scientific Books.

[28] Williams JLD 1975 Anatomical studies of the insect central nervous system: a ground-plan of the midbrain and an introduction to the central complex in the locust, *Schistocerca gregaria* (Orthoptera). J. Zool. 176, 67–86. (10.1111/j.1469-7998.1975.tb03188.x)

[29] Hanesch U, Fischbach KF, Heisenberg M 1989 Neuronal architecture of the central complex in *Drosophila melanogaster*. Cell Tissue Res. 257, 343–366. (10.1007/BF00261838)

[30] Ritzmann RE, Ridgel AL, Pollack AJ 2008 Multi-unit recording of antennal mechano-sensitive units in the central complex of the cockroach, *Blaberus discoidalis*. J. Comp. Physiol. A Neuroethol. Sens. Neural. Behav. Physiol. 194, 341–360. (10.1007/s00359-007-0310-2) 18180927

[31] Vitzthum H, Muller M, Homberg U 2002 Neurons of the central complex of the locust *Schistocerca gregaria* are sensitive to polarized light. J. Neurosci. 22, 1114–1125. 11826140 10.1523/JNEUROSCI.22-03-01114.2002 PMC6758510

[32] Heinze S, Reppert SM 2011 Sun compass integration of skylight cues in migratory monarch butterflies. Neuron 69, 345–358. (10.1016/j.neuron.2010.12.025) 21262471

[33] Phillips-Portillo J, Strausfeld NJ 2012 Representation of the brain's superior protocerebrum of the flesh fly, *Neobellieria bullata*, in the central body. J. Comp. Neurol. 520, 3070–3087. (10.1002/cne.23094) 22434505 PMC4876858

[34] Seelig JD, Jayaraman V 2015 Neural dynamics for landmark orientation and angular path integration. Nature 521, 186–191. (10.1038/nature14446) 25971509 PMC4704792

[35] Liu G, Seiler H, Wen A, Zars T, Ito K, Wolf R, Heisenberg M, Liu L 2006 Distinct memory traces for two visual features in the *Drosophila* brain. Nature 436, 551–556. (10.1038/nature04381) 16452971

[36] Ritzmann RE et al. 2012 Deciding which way to go: how do insects alter movements to negotiate barriers? Front. Neurosci. 6, 97 (10.3389/fnins.2012.00097) 22783160 PMC3390555

[37] Diaper DC et al. Submitted A lineage-related reciprocal inhibition circuitry for sensory-motor action selection. Nature Commun.

[38] Strausfeld NJ 2012 Arthropod brains: evolution, functional elegance and historical significance. Cambridge, MA: Harvard University Press.

[39] Strauss R, Heisenberg M 1993 A higher control center of locomotor behavior in the *Drosophila* brain. J. Neurosci. 5, 1852–1861. 10.1523/JNEUROSCI.13-05-01852.1993 PMC6576564 8478679

[40] Martin J, Faure P, Ernst R 2001 The power law distribution for walking-time intervals correlates with the ellipsoid-body in *Drosophila*. J. Neurogenet. 3–4, 205–219. (10.3109/01677060109167377) 12092904

[41] Sakura M, Lambrinos D, Labhart T 2007 Polarized skylight navigation in insects: model and electrophysiology of e-vector coding by neurons in the central complex. J. Neurophysiol. 99, 667–682. (10.1152/jn.00784.2007) 18057112

[42] Neuser K, Triphan T, Mronz M, Poeck B, Strauss R 2008 Analysis of a spatial orientation memory in *Drosophila*. Nature 453, 1244–1247. (10.1038/nature07003) 18509336

[43] Pan Y, Zhou Y, Guo C, Gong H, Gong Z, Liu L 2009 Differential roles of the fan-shaped body and the ellipsoid body in *Drosophila* visual pattern memory. Learn. Mem. 5, 289–295. (10.1101/lm.1331809) 19389914

[44] Lebestky T, Chang JS, Dankert H, Zelnik L, Kim YC, Han KA, Wolf FW, Perona P, Anderson DJ 2009 Two different forms of arousal in *Drosophila* are oppositely regulated by the dopamine D1 receptor ortholog DopR via distinct neural circuits. Neuron 64, 522–536. (10.1016/j.neuron.2009.09.031) 19945394 PMC2908595

[45] Heinze S, Gotthardt S, Homberg U 2009 Transformation of polarized light information in the central complex of the locust. J. Neurosci. 29, 11 783–11 793. (10.1523/JNEUROSCI.1870-09.2009) PMC6666666 19776265

[46] Kong EC et al. 2010 A pair of dopamine neurons target the D1-like dopamine receptor DopR in the central complex to promote ethanol-stimulated locomotion in *Drosophila*. PLoS One 5, e9954 (10.1371/journal.pone.0009954) 20376353 PMC2848596

[47] Bender JA, Pollack AJ, Ritzmann RE 2010 Neural activity in the central complex of the insect brain is linked to locomotor changes. Curr. Biol. 20, 921–926. (10.1016/j.cub.2010.03.054) 20451382

[48] Ofstad TA, Zuker CS, Reiser MB 2011 Visual place learning in *Drosophila melanogaster*. Nature 447, 204–207. (10.1038/nature10131) PMC3169673 21654803

[49] Guo P, Ritzmann RE 2013 Neural activity in the central complex of the cockroach brain is linked to turning behaviors. J. Exp. Biol. 216, 992–1002. (10.1242/jeb.080473) 23197098

[50] Seelig JD, Jayaraman V 2013 Feature detection and orientation tuning in the *Drosophila* central complex. Nature 503, 262–266. (10.1038/nature12601) 24107996 PMC3830704

[51] Kathman ND, Kesavan M, Ritzmann RE 2014 Encoding wide-field motion and direction in the central complex of the cockroach *Blaberus discoidalis*. J. Exp. Biol. 217, 4079–4090. (10.1242/jeb.112391) 25278467

[52] Young JM, Armstrong JD 2010 Structure of the adult central complex in *Drosophila*: organization of distinct neuronal subsets. J. Comp. Neurol. 518, 1500–1524. (10.1002/cne.22284) 20187142

[53] Kuntz S, Poeck B, Sokolowski MB, Strauss R 2012 The visual orientation memory of *Drosophila* requires Foraging (PKG) upstream of Ignorant (RSK2) in ring neurons of the central complex. Learn. Mem. 19, 337–340. (10.1101/lm.026369.112) 22815538 PMC3407938

[54] Urizar NL, Yang Z, Edenberg HJ, Davis RL 2007 *Drosophila* homer is required in a small set of neurons including the ellipsoid body for normal ethanol sensitivity and tolerance. J. Neurosci. 27, 4541–4551. (10.1523/JNEUROSCI.0305-07.2007) 17460067 PMC6672997

[55] Shih CT et al. 2015 Connectomics-based analysis of information flow in the *Drosophila* brain. Curr. Biol. 25, 1249–1258. (10.1016/j.cub.2015.03.021) 25866397

[56] Reiner A 2010 The conservative evolution of the vertebrate basal ganglia. In Handbook of basal ganglia structure and function (eds Steiner H, Tseng KY), pp. 29–62. Burlington, MA: Academic Press.

[57] Grillner S, Robertson B 2015 The basal ganglia downstream control of brainstem motor centres—an evolutionarily conserved strategy. Curr. Opin. Neurobiol. 12, 47–52. (10.1016/j.conb.2015.01.019) 25682058

[58] Polenova OA, Vesselkin NP 1993 Olfactory and nonolfactory projections in the river lamprey (*Lampetra fluviatilis*) telencephalon. J. Hirnforsch. 34, 261–279. 7693801

[59] Krout KE, Loewy AD, Westby GWM, Redgrave P 2001 Superior colliculus projections to midline and intralaminar thalamic nuclei of the rat. J. Comp. Neurol. 431, 198–216. (10.1002/1096-9861(20010305)431:2<198::AID-CNE1065>3.0.CO;2-8) 11170000

[60] McHaffie JG, Stanford TR, Stein BE, Coizet V, Redgrave P 2005 Subcortical loops through the basal ganglia. Trends Neurosci. 28, 401–407. (10.1016/j.tins.2005.06.006) 15982753

[61] Robertson B, Saitoh K, Menard A, Grillner S 2006 Afferents of the lamprey optic tectum with special reference to the GABA input: combined tracing and immunohistochemical study. J. Comp. Neurol. 499, 106–119. (10.1002/cne.21078) 16958107

[62] Chevalier G, Mana S 2000 Honey-comb like structure of the intermediate layers of the rat superior colliculus, with additional observations in several other mammals: AChE patterning. J. Comp. Neurol. 419, 137–153. (10.1002/(SICI)1096-9861(20000403)419:2<137::AID-CNE1>3.0.CO;2-6) 10722995

[63] Ocaña FM, Suryanarayana SM, Saitoh K, Kardamakis AA, Capantini L, Robertson B, Grillner S 2015 The lamprey pallium provides a blueprint of the mammalian motor projections from cortex. Curr. Biol. 25, 413–423. (10.1016/j.cub.2014.12.013) 25619762

[64] Stephenson-Jones M, Ericsson J, Robertson B, Grillner S 2012 Evolution of the basal ganglia: dual-output pathways conserved throughout vertebrate phylogeny. J. Comp. Neurol. 520, 2957–2973. (10.1002/cne.23087) 22351244

[65] Pérez-Fernández J, Stephenson-Jones M, Suryanarayana SM, Robertson B, Grillner S 2014 Evolutionarily conserved organization of the dopaminergic system in lamprey: SNc/VTA afferent and efferent connectivity and D2 receptor expression. J. Comp. Neurol. 522, 3775–3794. (10.1002/cne.23639) 24942187

[66] Redgrave P et al. 2010 Goal-directed and habitual control in the basal ganglia: implications for Parkinson's disease. Nat. Rev. Neurosci. 11, 760–772. (10.1038/nrn2915) 20944662 PMC3124757

[67] Nambu A 2011 Somatotopic organization of the primate basal ganglia. Front. Neuroanat. 5, 26 (10.3389/fnana.2011.00026) 21541304 PMC3082737

[68] Alexander GE, DeLong MR, Strick PL 1986 Parallel organization of functionally segregated circuits linking basal ganglia and cortex. Annu. Rev. Neurosci. 9, 357–381. (10.1146/annurev.ne.09.030186.002041) 3085570

[69] Redgrave P, Prescott TJ, Gurney K 1999 The basal ganglia: a vertebrate solution to the selection problem? Neuroscience 89, 1009–1023. (10.1016/S0306-4522(98)00319-4) 10362291

[70] Gurney KN, Prescott TJ, Redgrave P 2001 A computational model of action selection in the basal ganglia. I. A new functional anatomy. Biol. Cybern. 84, 401–410. (10.1007/PL00007984) 11417052

[71] Gurney KN, Prescott TJ, Redgrave P 2001 A computational model of action selection in the basal ganglia. II. Analysis and simulation of behaviour. Biol. Cybern. 84, 411–423. (10.1007/PL00007985) 11417053

[72] Ericsson J, Stephenson-Jones M, Pérez-Fernandez J, Robertson B, Silberberg G, Grillner S 2013 Dopamine differentially modulates the excitability of striatal neurons of the direct and indirect pathways in lamprey. J. Neurosci. 33, 8045–8054. (10.1523/JNEUROSCI.5881-12.2013) 23637194 PMC6618979

[73] Volkmann J, Daniels C, Witt K 2010 Neuropsychiatric effects of subthalamic neurostimulation in Parkinson disease. Nat. Rev. Neurol. 6, 487–498. (10.1038/nrneurol.2010.111) 20680036

[74] Bar-Gad I, Morris G, Bergman H 2003 Information processing, dimensionality reduction and reinforcement learning in the basal ganglia. Prog. Neurobiol 71, 439–473. (10.1016/j.pneurobio.2003.12.001) 15013228

[75] Gruber AJ, McDonald RJ 2012 Context, emotion, and the strategic pursuit of goals: interactions among multiple brain systems controlling motivated behavior. Front. Behav. Neurosci. 6, 50 (10.3389/fnbeh.2012.00050) 22876225 PMC3411069

[76] Mannella F, Gurney K, Baldassarre G 2013 The nucleus accumbens as a nexus between values and goals in goal-directed behavior: a review and a new hypothesis. Front. Behav. Neurosci. 7, e1–e29. (10.3389/fnbeh.2013.00135) PMC3805952 24167476

[77] Reig R, Silberberg G 2014 Multisensory integration in the mouse striatum. Neuron 83, 1200–1212. (10.1016/j.neuron.2014.07.033) 25155959 PMC4157575

[78] Saitoh K, Ménard A, Grillner S 2007 Tectal control of locomotion, steering, and eye movements in lamprey. J. Neurophysiol. 97, 3093–3108. (10.1152/jn.00639.2006) 17303814

[79] Wiener SI 1993 Spatial and behavioural correlates of striatal neurons in rats performing a self-initiated navigation task. J. Neurosci. 13, 3802–3817. 8366346 10.1523/JNEUROSCI.13-09-03802.1993 PMC6576451

[80] Kim N, Barter JW, Sukharnikova T, Yin HH 2014 Striatal firing rate reflects head movement velocity. Eur. J. Neurosci. 40, 3481–3490. (10.1111/ejn.12722) 25209171

[81] Barter JW, Li S, Sukharnikova T, Rossi MA, Bartholomew RA, Yin HH 2015 Basal ganglia outputs map instantaneous position coordinates during behavior. J. Neurosci. 35, 2703–2716. (10.1523/JNEUROSCI.3245-14.2015) 25673860 PMC4323537

[82] Saga Y, Hashimoto M, Tremblay L, Tanji J, Hoshi E 2013 Representation of spatial- and object-specific behavioral goals in the dorsal globus pallidus of monkeys during reaching movement. J. Neurosci. 33, 16 360–16 371. (10.1523/JNEUROSCI.1187-13.2013) PMC6618353 24107966

[83] Yin HH 2014 How the basal ganglia output generates behavior. Adv. Neurosci. 2014, 768313 (10.1155/2014/768313)

[84] Servan-Schreiber D, Printz H, Cohen JD 1990 A network model of catecholamine effects: gain, signal-to-noise ratio, and behavior. Science 249, 892–895. (10.1126/science.2392679) 2392679

[85] Grossberg S 1988 Nonlinear neural networks: principles, mechanisms, and architectures. Neural Netw. 1, 17–61. (10.1016/0893-6080(88)90021-4)

[86] Sussillo D 2014 Neural circuits as computational dynamical systems. Curr. Opin. Neurobiol. 25, 156–163. (10.1016/j.conb.2014.01.008) 24509098

[87] Baldassarre G, Mannella F, Fiore VG, Redgrave P, Gurney K, Mirolli M 2013 Intrinsically motivated action–outcome learning and goal-based action recall: a system-level bio-constrained computational model. Neural Netw. 41, 168–187. (10.1016/j.neunet.2012.09.015) 23098753

[88] Fiore VG, Sperati V, Mannella F, Mirolli M, Gurney K, Friston K, Dolan RJ, Baldassarre G 2014 Keep focussing: striatal dopamine multiple functions resolved in a single mechanism tested in a simulated humanoid robot. Front. Psychol. 5, 124 (10.3389/fpsyg.2014.00124) 24600422 PMC3930917

[89] Humphries MD, Stewart RD, Gurney KN 2006 A physiologically plausible model of action selection and oscillatory activity in the basal ganglia. J. Neurosci. 26, 12 921–12 942. (10.1523/JNEUROSCI.3486-06.2006) 17167083 PMC6674973

[90] Fiore VG, Rigoli F, Stenner MP, Zaehle T, Hirth F, Heinze HJ, Dolan RJ Submitted Changing pattern in the basal ganglia: motor switching under reduced dopaminergic drive. Sci. Rep. 10.1038/srep23327 PMC4804216 27004463

[91] Costa RM 2011 A selectionist account of de novo action learning. Curr. Opin. Neurobiol. 21, 579–586. (10.1016/j.conb.2011.05.004) 21641793

[92] Cohen JD, McClure SM, Yu AJ 2007 Should I stay or should I go? How the human brain manages the trade-off between exploitation and exploration. Phil. Trans. R. Soc. B 362, 933–942. (10.1098/rstb.2007.2098) 17395573 PMC2430007

[93] Grillner S, Hellgren J, Ménard A, Saitoh K, Wikström MA 2005 Mechanisms for selection of basic motor programs—roles for the striatum and pallidum. Trends Neurosci. 28, 364–370. (10.1016/j.tins.2005.05.004) 15935487

[94] Kravitz AV, Freeze BS, Parker PR, Kay K, Thwin MT, Deisseroth K, Kreitzer AC 2010 Regulation of parkinsonian motor behaviours by optogenetic control of basal ganglia circuitry. Nature 466, 622–626. (10.1038/nature09159) 20613723 PMC3552484

[95] Cui G, Jun SB, Jin X, Pham MD, Vogel SS, Lovinger DM, Costa RM 2013 Concurrent activation of striatal direct and indirect pathways during action initiation. Nature 494, 238–242. (10.1038/nature11846) 23354054 PMC4039389

[96] Isomura Y, Takekawa T, Harukuni R, Handa T, Aizawa H, Takada M, Fukai T 2013 Reward-modulated motor information in identified striatum neurons. J. Neurosci. 33, 10 209–10 220. (10.1523/JNEUROSCI.0381-13.2013) PMC6618603 23785137

[97] Frank MJ 2006 Hold your horses: a dynamic computational role for the sub-thalamic nucleus in decision making. Neural Netw. 19, 1120–1136. (10.1016/j.neunet.2006.03.006) 16945502

[98] Tecuapetla F, Matias S, Dugue GP, Mainen ZF, Costa RM 2014 Balanced activity in basal ganglia projection pathways is critical for contraversive movements. Nat. Commun. 5, 4315 (10.1038/ncomms5315) 25002180 PMC4102112

[99] Pielage J, Steffes G, Lau DC, Parente BA, Crews ST, Strauss R, Klämbt C 2002 Novel behavioural and developmental defects associated with *Drosophila* *single-minded*. Dev. Biol. 249, 283–299. (10.1006/dbio.2002.0770) 12221007

[100] Freeze BS, Kravitz AV, Hammack N, Berke JD, Kreitzer AC 2013 Control of basal ganglia output by direct and indirect pathway projection neurons. J. Neurosci. 33, 18 531–18 539. (10.1523/JNEUROSCI.1278-13.2013) PMC3834057 24259575

[101] Montague PR, Dayan P, Sejnowski TJ 1996 A framework for mesencephalic dopamine systems based on predictive Hebbian learning. J. Neurosci. 16, 1936–1947. 8774460 10.1523/JNEUROSCI.16-05-01936.1996 PMC6578666

[102] Schultz W, Dayan P, Montague PR 1997 A neural substrate of prediction and reward. Science 275, 1593–1599. (10.1126/science.275.5306.1593) 9054347

[103] Waddell S 2010 Dopamine reveals neural circuit mechanisms of fly memory. Trends Neurosci. 33, 457–464. (10.1016/j.tins.2010.07.001) 20701984 PMC2947577

[104] Waddell S 2013 Reinforcement signaling in *Drosophila*; dopamine does it all after all. Curr. Opin. Neurobiol. 23, 324–329. (10.1016/j.conb.2013.01.005) 23391527 PMC3887340

[105] Gurney KN, Humphries MD, Redgrave P 2015 A new framework for cortico-striatal plasticity: behavioural theory meets *in vitro* data at the reinforcement–action interface. PLoS Biol. 13, e1002034 (10.1371/journal.pbio.1002034) 25562526 PMC4285402

[106] Jin X, Costa RM 2015 Shaping action sequences in basal ganglia circuits. Curr. Opin. Neurobiol. 33, 188–196. (10.1016/j.conb.2015.06.011) 26189204 PMC4523429

[107] Seger CA, Spiering BJ 2011 A critical review of habit learning and the basal ganglia. Front. Syst. Neurosci. 5, 66 (10.3389/fnsys.2011.00066) 21909324 PMC3163829

[108] Haluk DM, Floresco SB 2009 Ventral striatal dopamine modulation of different forms of behavioral flexibility. Neuropsychopharmacology 34, 2041–2052. (10.1038/npp.2009.21) 19262467

[109] Lorenz K, Tinbergen N 1937 Taxis und Instinkthandlung in der Eirollbewegung der Graugans. Zeitschr. Tierphysiol. 2, 1–29. (10.1111/j.1439-0310.1939.tb01558.x)

